# Automated qualitative and quantitative assessment of posterior capsule opacification by Automated Quantification of After-Cataract II (AQUA II) system

**DOI:** 10.1186/s12886-019-1116-z

**Published:** 2019-05-17

**Authors:** Martin Kronschläger, Hannes Siegl, Axel Pinz, Christoph Feichtenhofer, Wolf Buehl, Nino Hirnschall, Oliver Findl

**Affiliations:** 1 Department of Ophthalmology, Vienna Institute for Research in Ocular Surgery (VIROS), Hanusch Hospital, Heinrich-Collin Str. 30, Vienna, Austria; 2 Department of Ophthalmology, Medical University of Vienna, Vienna, Austria; 3 Institute of Electrical Measurement and Measurement Signal Processing, Graz Technical University, Graz, Austria

**Keywords:** Automated quantification of after-cataract, AQUA, Posterior capsule opacification, Retroillumination image

## Abstract

**Background:**

The present study aims to investigate an automated qualitative and quantitative assessment system (Automated Quantification of After-Cataract [AQUA II]) of posterior capsule opacification (PCO) in high-resolution digital retroillumination images and consequently reduce observer bias and increase accuracy of PCO grading.

**Methods:**

A data set of 100 eyes with no to severe PCO was analysed. Ten eyes were consecutively photographed twice and ten images were rotated to give a total of 120 images for PCO assessment. Validity was determined by including subjective grading and repeatability was determined by evaluating the 20 additional images. Evaluation of posterior capsular opacification (EPCO), posterior capsule opacity (POCO) and AQUA I methods were included for comparative analysis of the data.

**Results:**

The system developed proved to classify six types of PCO. Validity was confirmed by a Pearson correlation coefficient of *r* = 0.95 (EPCO *r* = 0.93; POCO *r* = 0.72 and AQUA I *r* = 0.94). Repeatability was better in AQUA II (95% confidence interval [CI] for mean difference: 0.5 ± 1.2) than in subjective grading (95% CI for mean difference: 0.6 ± 1.7), in EPCO grading (95% CI for mean difference: − 0.2 ± 1.5), in POCO grading (95% CI for mean difference: 1.6 ± 2.7) and in AQUA I (95% CI for mean difference: − 1.1 ± 1.9).

**Conclusions:**

AQUA II is a system that for the first time not only objectively quantifies PCO, but also qualitatively assesses PCO in an automated manner with texture classification. AQUA II showed an excellent validity and repeatability.

## Background

Cataract is still the leading cause of blindness in the world [1] and the only therapy is cataract surgery. The most common complication of cataract surgery is posterior capsule opacification (PCO) [2]. PCO induces forward light scattering and consequently reduces visual acuity. Incidence of PCO varies between studies from as low as < 5% [3] to as high as 50% [4]. Therefore, techniques to evaluate PCO and to study the results of PCO preventing methods are needed.

PCO develops from lens epithelial cells (LEC) remaining in the capsular bag after cataract surgery. Two basic types of PCO have been described, i.e., firstly regeneratory PCO including honeycomb, PCO plates and Elschnig pearls and, secondly fibrotic PCO. Regeneratory PCO is thought to evolve from LECs in the pre-equatorial zone and typically begins with a honeycomb form until maturing into pearls. Fibrotic PCO, however, originates from the LECs residing in the anterior capsule. PCO may consist of either regeneratory PCO or fibrotic PCO or a combination of both.

One option to study PCO is to take digital retroillumination photographs of the posterior capsule. To analyse the digital photographs several pattern analysis systems are available. In addition to a subjective analysis using a score from 0 to 10, evaluation of posterior capsular opacification (EPCO) introduced by Tetz et al. [5], is an attempt to better quantify the grading. After manual segmentation, segments are graded subjectively from mild 1 to severe 4. The EPCO score results from summing up the graded segments. Contrary to the qualitative grading of EPCO, posterior capsule opacity (POCO) quantitatively measures the area affected by PCO [6]. POCO, however, provides no qualitative information. A further method to quantitatively measure PCO is the AQUA I method [7], which was developed by our group. Scores of the AQUA I program represents grey level co-occurrence matrix entropy, a measure of inhomogeneity. A global AQUA I score, however, gives no information on localization of the PCO. To overcome the limitations of POCO and AQUA I regarding qualitative analysis, we composed AQUA II, a fully automated qualitative and quantitative measure system. This study aims to present qualitative analysis with AQUA II. Moreover, PCO quantification results of AQUA II are compared to subjective grading, EPCO, POCO and AQUA I scores.

## Methods

Qualitative analysis of PCO images were performed followed by computing of total PCO scores by AQUA II system. A correlation coefficient between AQUA II and a subjective grading is calculated. Further correlation coefficients between EPCO and subjective grading, between POCO and subjective scoring and between AQUA I and subjective grading are determined. Finally, repeatability of AQUA II is evaluated.

### Digital image data set

A set of 100 digital images of eyes of 100 patients with an even distribution of mild to severe PCO including clear capsules were already created for our previous study [8] from a pool of images for quality assurance measures in 2002, and is referred to as the Vienna Eye Clinic (VEC) data set. Patient identifying information were not accessible. Images of the VEC data set were acquired by an optical system consisting of a Zeiss 30 slit lamp for observation and imaging, a Zeiss retrolux illumination module with illumination provided by a Zeiss anterior segment flash pack through a fiber-optic cable, and beam splitters. A high signal-to-noise ratio and light sensitivity were achieved with a Kodak NC 2000 digital camera containing a 16.0 mm X 21.0 mm charge coupled device (CCD) chip. The CCD has a geometric resolution of 1268 pixels X 1012 pixels and a radiometric resolution of 36 bits (RGB). Images were further imported to Adobe Photoshop® 5.5 and processed as TIFF files.

The region of interest (ROI), i.e., the posterior capsule, was defined as central 4.0 mm diameter of the IOL not containing any part of the anterior capsule. The ROI was extracted by Adobe Photoshop and analyzed by AQUA II, subjective grading, EPCO, POCO and AQUA I.

To assess repeatability, in addition to the 100 images of 100 eyes, 2 photographs were taken consecutively in 10 cases and another 10 images were duplicated and rotated in Adobe Photoshop. In total, the VEC data set included 120 images randomly ordered. None of the examiners either subjectively scoring or operating AQUA II, EPCO, POCO or AQUA I were informed about the purpose of the 20 additional images.

### AQUA II

For AQUA I [7], we used a global texture measure based on the gray-level co-occurrence matrix (GLCM) [9]. As the results seemed promising, the method was refined using locally computed texture features [10] which means that texture features were computed for a defined local neighborhood of every pixel in order to perform texture-based segmentation into six classes (Table 1) with a PCO grading weight *w*_*i*_ from 0 to 4.

**Table 1:** Six classes and weights used for PCO assessment in AQUA II

PCO Assesment		
Texture characteristics	Color code	weight
clear	black	0
honeycomb A	cyan	0.8
honeycomb B	blue	1
plate	green	2
pearl plate	yellow	3
Elschnig pearls	red	4

Four approaches were applied to compute these local texture features: (1) GLCM; (2) first order features [11]; (3) a set of statistical features obtained from Gabor filter responses [12]; and (4) fractal features [13]. In extensive experiments using a combination of feature selection [14] and a Bayes classifier, the influence of scale, preprocessing and other parameters was inspected to obtain the best feature subset for classification of the image data. The global score for the PCO assessment was computed by Eq. 1 where *p*_*n*_ denotes the number of pixels assigned class n.

1 $$ AQUA\  II=\frac{\sum_{n=1}^6{p}_n\ast {w}_n}{\sum_{n=1}^6{p}_n} $$

**Fig. 1:**
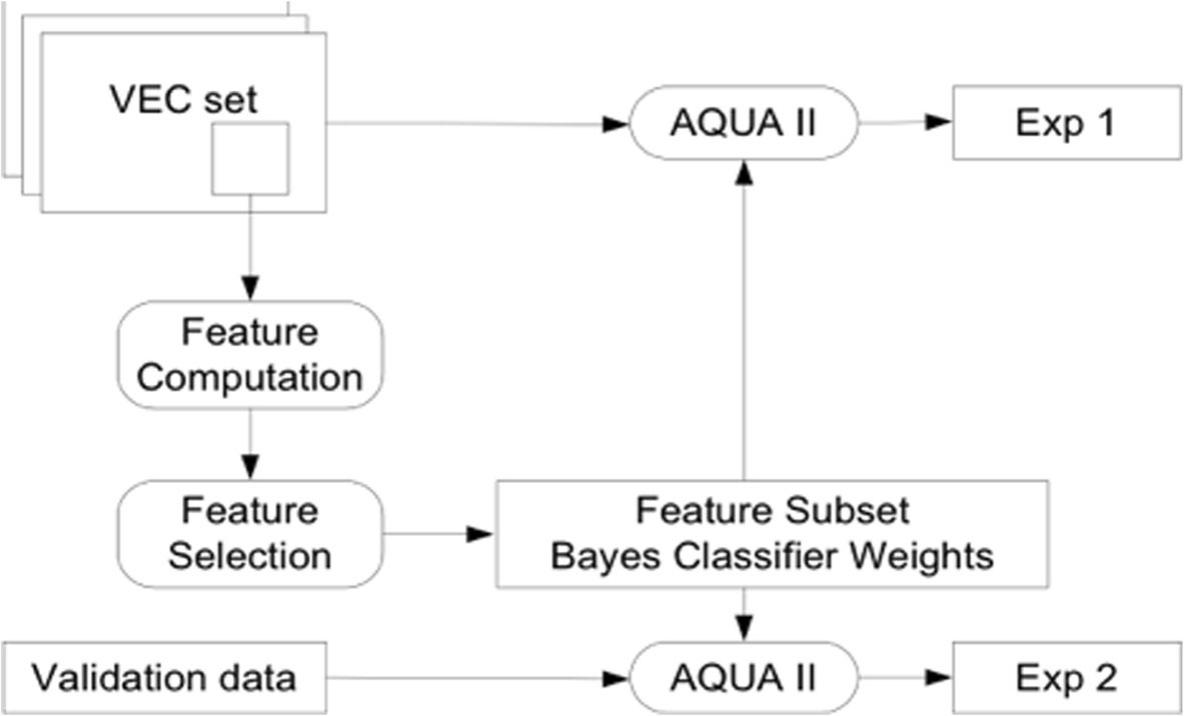
Experimental setup for selecting feature subset and grading. The training data (VEC, Vienna Eye Clinic) were the same data used for the AQUA I method

The resulting classification engine was validated against an additional set of 20 images with manual grading (Fig. 1). All experiments were performed with Matlab using the Imaging Processing Toolbox. Full technical details are provided in [10].

### Subjective analysis

A subjective score from 0 (clear capsule) to 10 (most intense PCO possible) was applied. Four experts graded the entire VEC data set 2 times with a pause of 1 week between the grading. Similarly, 3 student examiners graded the VEC data set. Finally, a group of 3 experts evaluated the results of the grading of both the experts and the students and performed a grading together. The data of this grading were taken from Findl et al. [8].

### Qualitative analysis

The VEC data set was analysed with AQUA II. Color coding used for the output images is given in Table 1. Moreover, images were analysed with EPCO and POCO to compare qualitative output.

### Validity

To assess the validity of the AQUA II system, the results of the subjective analysis of the VEC data set were compared to the scores generated by the AQUA II system.

### Repeatability

To evaluate repeatability, data of 10 rotated images and 10 consecutively photographed eyes were used. The data set included equal numbers of eyes exhibiting mild, moderate, and severe opacification.

### Statistical analysis

For statistical assessment, scores of both the subjective analysis and the AQUA II system were converted to scores from 0 to 100. All data were statistically evaluated with Excel 2016. The Pearson correlation coefficient (r) was calculated to test validity. To analyze repeatability, the 95% confidence interval [CI] for mean difference of 2 measurements was determined and the coefficient of repeatability was calculated. The confidence coefficient was set to 0.95, considering the sample size, and the expected precision of estimates, respectively.

## Results

### Qualitative analysis

**Fig. 2:**
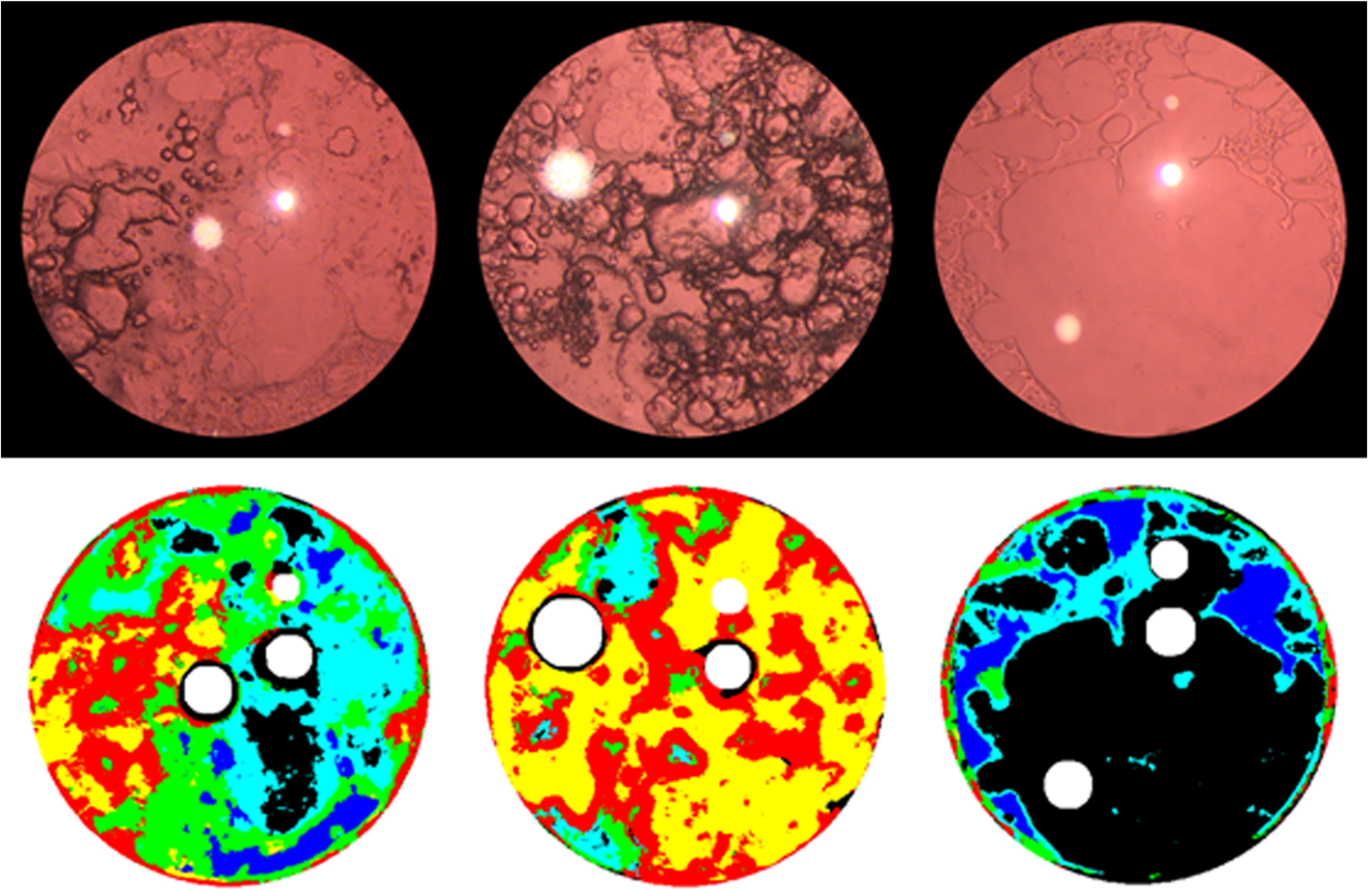
Three representative cases of qualitative analysis with AQUA II. Texture characteristics are color coded with clear lens (black), honeycomb A (cyan), honeycomb B (blue), plate (green), pearl plate (yellow) and Elschnig pearls (red)

**Fig. 3:**
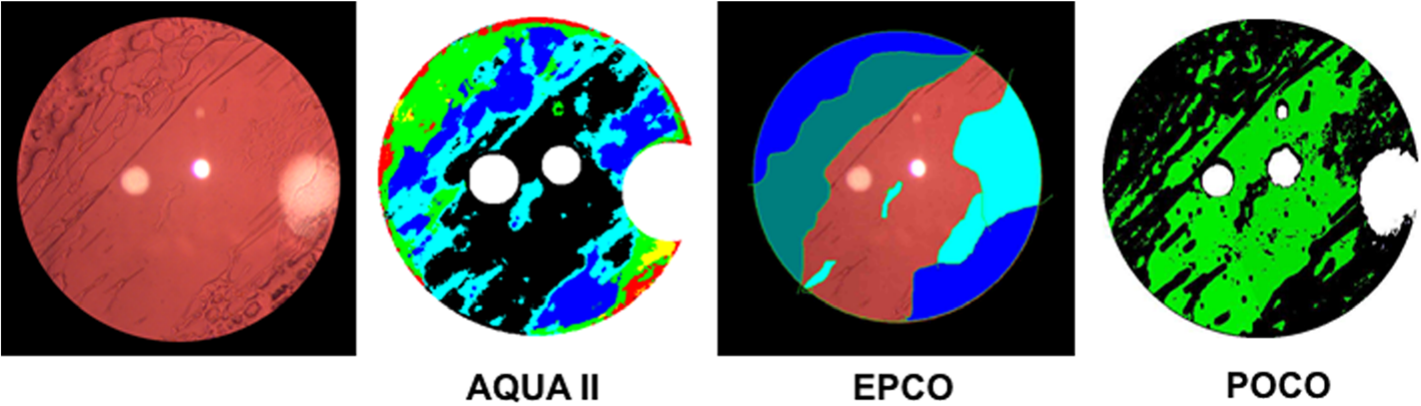
Qualitative comparison between digital image, AQUA II, EPCO (red = no PCO, cyan = mild PCO, green = moderate PCO, blue = severe PCO) and POCO (black = PCO, green = no PCO)

Contours of PCO correlated relatively well between AQUA II output images and the digital image set. Different textures of the PCO were well classified in AQUA II output images with the color code given (Fig. 2). Further, delineation lines of PCO correlated well between AQUA II output images, EPCO output images and POCO output images (Fig. 3).

### Validity

**Fig. 4:**
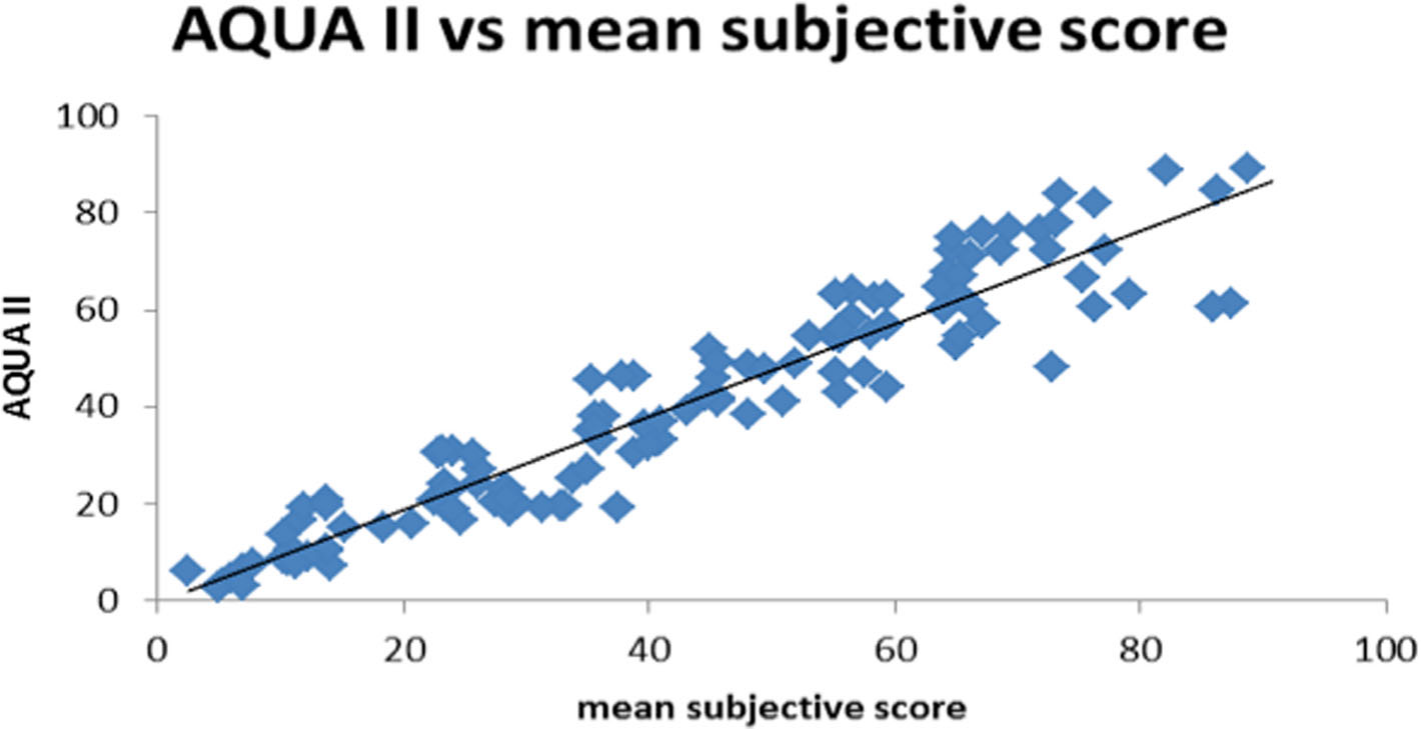
Correlation between mean subjective score and AQUA II. The trendline is a linear least mean square fit (*r* = 0.95)

**Fig. 5:**
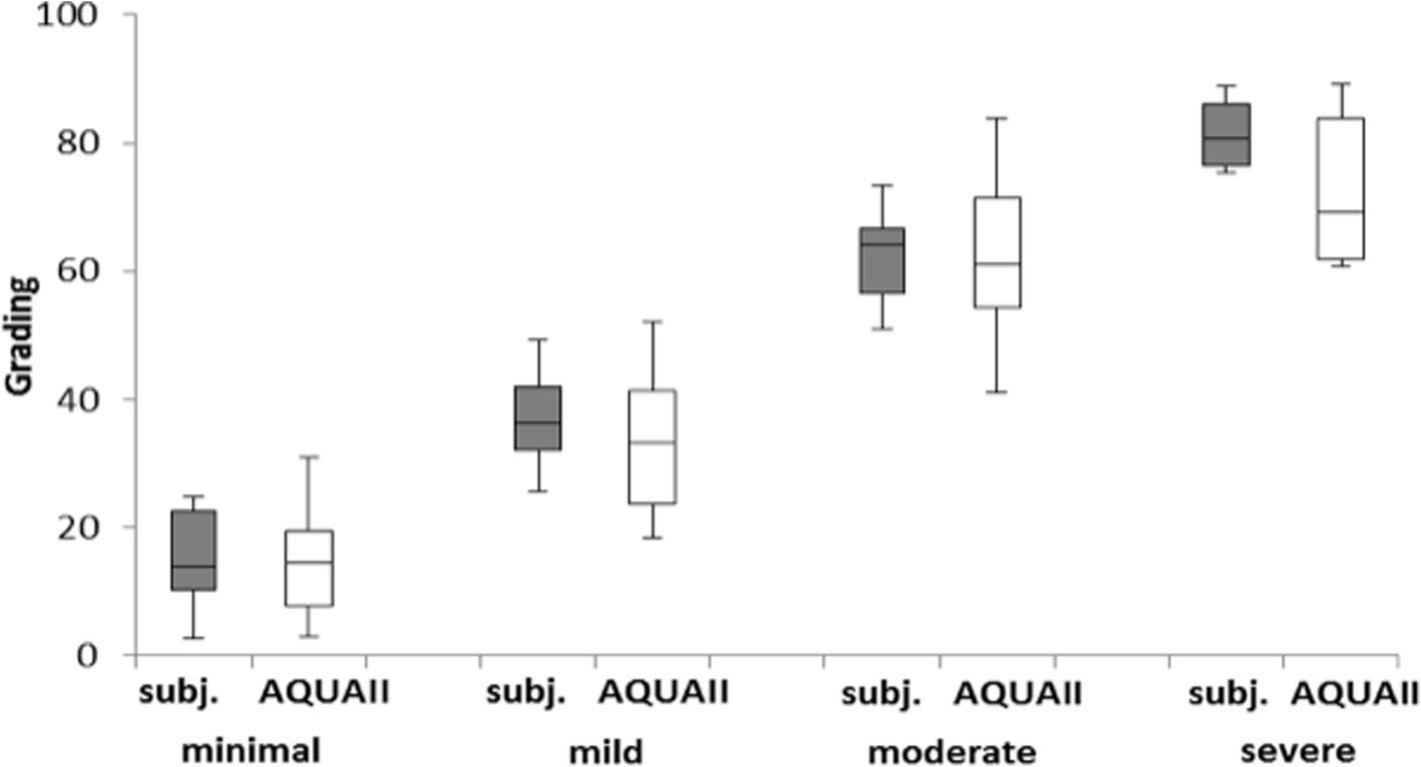
Box plots for subjective (subj.) vs AQUA II grading for four different PCO severity groups

The AQUA II derived measure of opacification and the grading scores of the subjective analysis showed excellent agreement, when the means of the clinical scores were correlated with the values from the software, *r* = 0.95. The results are shown in Fig. 4. Furthermore, an analysis in box plots is presented in Fig. 5.

Additionally, correlation between EPCO and subjective grading resulted in *r* = 0.93, correlation between POCO and subjective grading resulted in *r* = 0.72 and correlation between AQUA I and subjective grading resulted in *r* = 0.94.

### Repeatability

Mean difference of the grading for AQUA II, including all 10 rotated images and 10 consecutively photographed eyes, was 0.5 with a 95% confidence interval (CI) of ±1.2. The coefficient of repeatability (CR) was calculated to 5.1.

In comparison, subjective analysis of the same data set resulted in a mean difference of 0.6 with a 95% CI of ±1.7. The CR was 7.7. Analysis with EPCO resulted in a mean difference of − 0.2 with a 95% CI of ±1.5 and a CR of 6.8. Analysis with POCO resulted in a mean difference of 1.6 with a 95% CI of ±2.7 and a CR of 12.2. Analysis with AQUA I resulted in a mean difference of − 1.1 ± 1.9. and a CR of 8.4.

**Fig. 6:**
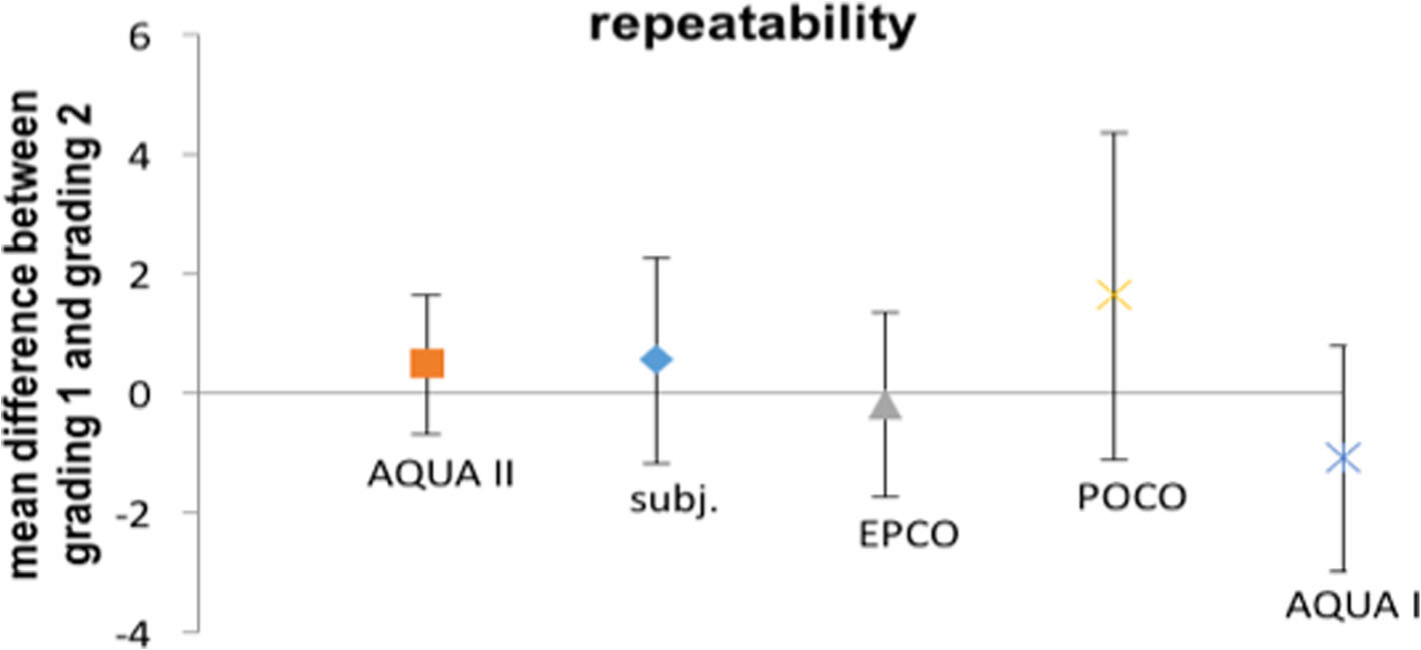
Comparison of repeatability of subjective (subj.) grading, AQUA II, EPCO, POCO and AQUA I. Bars are 95% CI

Mean differences and CI for all applied methods are given in Fig. 6.

## Discussion

In the current study the AQUA II system was used for qualitatively and quantitatively analyzing PCO from the VEC data set. The VEC data set was created in 2002 and consists of images of 100 eyes with different degrees of PCO. Findl et al. already used the VEC data set for comparison of 4 methods (subjective analysis, EPCO, POCO and AQUA I) of PCO quantification [8]. Averaged subjective data from this study [8] were used to show validity of AQUA II and to compare repeatability in the current study. Moreover, Findl et al. measured the correlation between subjective analysis and EPCO (*r* = 0.93), subjective analysis and POCO (*r* = 0.72) and subjective analysis and AQUA I (*r* = 0.94) [8]. We analysed the same data (VEC data set) with AQUA II and achieved a correlation of *r* = 0.95. This represents the highest correlation between a subjective grading and those objective measure methods included. It might be interesting for future investigations to compare AQUA II to OCT [15] and Scheimpflug [16] based methods.

The goal of automation is to reduce observer bias and to increase accuracy. Ranging from subjective to objective various qualitative assessment methods of PCO images are available. EPCO is a subjective method with 4 grades (Fig. 3). POCOman is a semi - objective assessment and correlates well with POCO [17]. POCO is an automated objective assessment tool, however, lacking classification of PCO textures (Fig. 3). AQUA II represents an automated objective assessment tool for PCO classifying PCO in 6 types (Table 1). The main problem of the VEC data set images are the Purkinje reflexes generated from axial illumination leading to lost data. Findl et al. described the removal of those reflexes by image – fusion [18]. However, at the time of VEC data set collection this method was yet not published. Nevertheless, to compare AQUA II to already existing data of subjective scoring, AQUA I, POCO and EPCO grading, we chose to analyse the VEC data set.

We found when evaluating validity, that especially in severe PCO (Fig. 5) AQUA II underestimates PCO expression. This may result from the fact that the weighting of classes was set arbitrarily and that the location of PCO structures does not affect the grading. In clinics, peripheral PCO is sometimes not even perceived by the patient. However, this very important issue of PCO location is also not covered by other retroillumination based analyzing methods. A minor weakness of our system is that it is not able to robustly detect large single pearls, because these are rather defined by their boundaries and stage than they are defined by their texture. Active contours may solve this problem. A critical issue in image analysis is defining the region of interest. We chose to use the central 4.0 mm of the IOL as objective measure. The automated POCO system protocol includes a subjective step in defining the region of interest by the examiner as the posterior capsule that is behind the optic of the IOL and is not obscured by the anterior capsule.

The evaluation of repeatability (Fig. 6) shows that AQUA II is superior to all included grading methods (subjective grading, EPCO, POCO and AQUA I). In consecutive images, variations due to slight changes in fixation and consequently different degrees of illumination and variation in image section may occur. In rotated images the image section and illumination are constant. AQUA II proved to be robust in both conditions.

On clinical level, AQUA II offers an effective, objective, automated and reproducible method for qualitative and quantitative PCO assessment. Moreover, contrast sensitivity is depends on the type of PCO [19]. AQUA II would allow correlation of tissue classification and affected area with contrast sensitivity. In addition, PCO leads to higher order optical aberrations [20]. AQUA II tissue classification maps may be useful in explaining higher order aberration maps of the eye.

## Conclusion

The AQUA II system described in this study is a novel fully automated and repeatable method that not only quantifies PCO accurately, but for the first time offers an objective texture dependent method for qualitative PCO analysis. Therefore, AQUA II represents an important improvement to AQUA I and should help in experimental and clinical trials to further investigate PCO.

## Abbreviations

AQUA: Automated Quantification of After-Cataract
CCD: Charge coupled device
CI: Confidence interval
CR: Coefficient of repeatability
EPCO: Evaluation of posterior capsular opacification
GLCM: Gray-level co-occurrence matrix
IOL: Intraocular lens
LEC: Lens epithelial cells
OCT: Optical coherence tomography
PCO: Posterior capsule opacification
POCO: Posterior capsule opacity evaluation program
VEC: Vienna Eye Clinic
VIROS: Vienna Institute for Research in Ocular Surgery
